# Risk factors associated with radiolucent foreign body inhalation in adults: a 10-year retrospective cohort study

**DOI:** 10.1186/s12931-022-02165-9

**Published:** 2022-09-10

**Authors:** Xiaofan Liu, Fang Ni, Tao Guo, Fangfang Jiang, Yan Jiang, Cheng Song, Mingli Yuan, Zhaowu Tao, Mingxin Ye, Junjie Xu, Ying Wang, Qiong Qian, Yi Hu, Yihua Wang

**Affiliations:** 1 Department of Pulmonary and Critical Care Medicine, The Central Hospital of Wuhan, Tongji Medical College, Huazhong University of Science and Technology, Wuhan, 430014 Hubei China; 2 Biological Sciences, Faculty of Environmental and Life Sciences, University of Southampton, Southampton, SO17 1BJ UK; 3 Department of Mathematical Sciences, Faculty of Social Sciences, University of Southampton, Southampton, SO17 1BJ UK; 4 Department of Radiology, The Central Hospital of Wuhan, Tongji Medical College, Huazhong University of Science and Technology, Wuhan, 430014 Hubei China; 5 Institute for Life Sciences, University of Southampton, Southampton, SO17 1BJ UK; 6 NIHR Southampton Biomedical Research Centre, University Hospital Southampton, Southampton, SO16 6YD UK

**Keywords:** Foreign body aspiration, Radiolucent, radiopaque, Multi-detector Computed Tomography (MDCT), Flexible bronchoscopy, Adult

## Abstract

**Background:**

Foreign body aspiration (FBA) is a serious condition with high morbidity and mortality rates. Although chest radiography is generally the first radiologic modality used in diagnosis, a substantial percentage of foreign bodies are radiolucent in adults with diagnosis challenging.

**Methods:**

Retrospective review of adult patients with FBA diagnosed by flexible electronic bronchoscopy from 2012 to 2022 collecting demographics, history, hospital presentation, radiographic, and operative details. Risk factors associated with radiolucent foreign body inhalation in adults were explored using appropriate statistical methods.

**Results:**

Between 1 January 2012 and 1 January 2022, 114 adult patients diagnosed with FBA were enrolled. The median age of participants was 65 years (IQR 52–74). Multidetector computed tomography (MDCT) examinations identified 28 cases (25%) showing direct visualization of the foreign body (defined as the *radiopaque* group) and 86 cases (75%) in the *radiolucent* group. Multivariable stepwise linear regression analysis showed increased odds of radiolucent foreign body inhalation in adults associated with pneumonic patches in MDCT (OR 6.99; 95% CI 1.80–27.22; *P* = 0.005) and plants/meat foreign bodies (OR 6.17; 95% CI 1.12–33.96; *P* = 0.04). A witnessed choking history (OR 0.02; 95% CI 0–0.14; *P* < 0.001) was a protective factor of radiolucent foreign body inhalation in adults.

**Conclusions:**

Unlike radiopaque FBA, in those presenting with a suspected radiolucent foreign body aspiration, the diagnosis is far more challenging. Risk factors such as lacking a choking history, non-resolving pneumonia (pneumonic patches) in MDCT findings, and plants/meat foreign bodies may help in the early diagnosis of radiolucent foreign body inhalation in adults. Further prospective multicenter studies should be conducted to validate the findings.

**Supplementary Information:**

The online version contains supplementary material available at 10.1186/s12931-022-02165-9.

## Background

Foreign body aspiration (FBA) is an uncommon but potentially life-threatening event [1, 2]. Symptoms typically consist of a choking event followed by cough and dyspnea [3, 4]. Although both adults and children tend to inhale foreign bodies, it is more common in children than in adults [4]. Several observational studies report a low rate of FBA in adults (0.66 per 100,000) [5–7]. However, the presentations in adults are inconsistent and symptoms may mimic more chronic lung diseases such as asthma or chronic obstructive pulmonary disease [6, 8–10]. In addition, many cases of FBA in adults lack a history of choking and are often with no direct signs of airway foreign body in chest radiographs [4, 11].

The initial clues for FBA in adults are usually obscure or indirect [11, 12]. Reports have indicated that only 25–38% of adult patients who are found to have a lower airway foreign body remember a choking event [13, 14]. This is supported by a systematic literature review showing that often a history of aspiration is lacking in adults with FBA [15]. Although chest radiography is the primary imaging modality used to identify a foreign body in the lower airway, a study from Sehgal and colleagues showed that only 24.6% of foreign body inhalation in the adult population has the radiological manifestations of foreign body aspiration and direct visualization of the foreign body in the case of radiopaque foreign bodies [5]. Unlike radiopaque FBA, in those presenting with a suspected radiolucent foreign body aspiration, the diagnosis is far more challenging [16–18]. When a diagnosis is not established immediately, retained foreign bodies may lead to recurrent pneumonia, recurrent hemoptysis, bronchiectasis, or other complications [5].

In this study, we aimed to analyze retrospectively to determine risk factors that help in the early diagnosis of radiolucent foreign body inhalation in adults.

## Study design and methods

### Participants

All methods were carried out following relevant guidelines and regulations. With approval from the Ethics Committee of the Central Hospital of Wuhan, Hubei, China (Approval No.: WHZXKYL2022-052), a retrospective review was conducted at the Department of Pulmonary and Critical Care Medicine in the Central Hospital of Wuhan. All adult patients (age > 18 years) diagnosed with FBA from 1 January 2012 to 1 January 2022 were enrolled. They all underwent multi-detector Computed Tomography (MDCT) examinations followed by flexible bronchoscopy (details in Additional file 1: Methods).

### Measures

Patient demographics, pre-hospital history, symptoms, radiographic presentations, operative details, bronchoscopic findings, and clinical course were collected from the medical records.

Patients underwent chest MDCT examinations in the supine position and with breath-holding following inspiration (GE Healthcare Optima) from the level of the thoracic inlet to the dome of the diaphragm. The CT examinations were performed with a rotation time of 0.6 s, a pitch ratio of 1.75, tube voltage of 120 kV, tube current adjust automatically, and a slice thickness of 1.25 mm for a whole chest scan. MDCT images were reconstructed with a 1.25-mm slice thickness on the workstation (GE ADW 2.0). These reconstructed axial images were processed. On the 3D page, the reconstructed multiplanar reformation (MPR) images were evaluated in the axial, sagittal, and coronal planes. MDCT examinations showing direct visualization of the foreign body are defined as *radiopaque* cases, while *radiolucent* cases are those foreign bodies that failed to be identified by 2 experienced radiologists. They reviewed the images independently in a consistent manner, with a conclusion reached by consensus when there was a discrepancy.

### Statistical analysis

Categorical variables were expressed as n (%) and compared by χ2 test or Fisher's exact test if appropriate. Continuous variables were expressed as median (IQR, interquartile range), and compared using a two-sample *t*-test, Welch's two-sample *t*-test, or Mann–Whitney *U* test, if appropriate. Normality of distribution was assessed using the Shapiro–Wilk test. Because of the sample size, measurable variables with significant differences between groups were considered in subsequent univariate and multivariable logistic regression analyses [19–24]. A multivariable binary logistic regression analysis (backward stepwise method) was performed to identify the independent risk factors associated with radiolucent foreign body inhalation in adults. Cut off point was determined by the receiver operating characteristic curve. *P* values less than 0.05 were considered statistically significant. All data analyses and graphs were done in R (version 4.1.3).

## Results

### Patient characteristics

As shown in Table 1, between 1 January 2012 and 1 January 2022, 114 adult patients diagnosed with FBA were enrolled, of whom 71 were male (62%) and 43 females (38%), with a median age of 65 years (IQR 52 ~ 74). A witnessed choking history was observed in 19 (17%) patients. 49 cases (43%) were with a length of disease course of more than 60 days. 12 patients (11%) had been admitted to ICU, mainly due to airway obstruction, respiratory failure, and multiple organ dysfunction. 64 cases (56%) had been misdiagnosed as pneumonia and 5 cases (4%) as lung cancer.

**Table 1:** Patient characteristics

	All (n = 114)	Radiopaque (n = 28)	Radiolucent (n = 86)	P-value
Age, years				
	65 (52–74)	66 (52–75)	62 (54–68)	0.65
Sex				
Female	43 (38%)	10 (36%)	33 (38%)	0.98
Male	71 (62%)	18 (64%)	53 (62%)	
Witnessed choking				
Yes	19 (17%)	17 (61%)	2 (2%)	< 0.001*
No	95 (83%)	11 (39%)	84 (93%)	
Length of disease course, days				
	30 (9–150)	7 (1–30)	60 (14–150)	< 0.001*
< 60	65 (57%)	23 (82%)	42 (49%)	0.004*
≥ 60	49 (43%)	5 (18%)	44 (51%)	
Experience in ICU				
Yes	12 (11%)	0 (0%)	12 (14%)	0.04*
No	102 (89%)	28 (100%)	74 (86%)	
Symptoms				
Coughing	103 (90%)	25 (89%)	78 (91%)	1
Purulent sputum	84 (74%)	21 (75%)	63 (73%)	1
Chest tightness	49 (43%)	13 (46%)	36 (42%)	0.84
Hemoptysis	25 (22%)	10 (36%)	15 (17%)	0.08
Fever	9 (8%)	0 (0%)	9 (10%)	0.11
Dyspnea	6 (5%)	1 (4%)	5 (6%)	1
Chest pain	5 (4%)	0 (0%)	5 (6%)	0.33
Disturbance of consciousness	1 (1%)	1 (4%)	0 (0%)	0.25
Physical examination				
Rales	24 (21%)	4 (14%)	20 (23%)	0.46
Wheezing	6 (5%)	1 (4%)	5 (6%)	1
Complications				
Chronic diseases requiring daily medication	59 (52%)	17 (61%)	42 (49%)	0.38
Chronic respiratory disease	30 (26%)	8 (29%)	22 (26%)	0.95
Post-stroke dysphagia	18 (16%)	4 (14%)	14 (16%)	1
Alzheimer's disease	3 (3%)	2 (7%)	1 (1%)	0.15
Throat surgery/diseases	2 (2%)	1 (4%)	1 (1%)	0.43
Dental operation	1 (1%)	1 (4%)	0 (0%)	0.25
Misdiagnosis				
Pneumonia	64 (56%)	7 (25%)	57 (66%)	< 0.001*
Cancer	5 (4%)	0 (0%)	5 (6%)	0.33

Data are median (IQR) or n (%). *P-value < 0.05 with statistical significance

80 patients (70%) had complications with 59 patients (52%) had a variety of chronic diseases requiring daily medication, including hypertension, coronary disease, and type 2 diabetes mellitus. 30 patients (26%) suffered chronic respiratory diseases presenting cough, expectoration, and dyspnea. 18 patients (16%) had post-stroke dysphagia, 3 patients (3%) with Alzheimer's disease, 2 patients (2%) with a history of throat surgery, and 1 patient with FBA during the dental operation.

For symptoms, coughing was the most common presentation of FBA (103 cases, 90%). Other presentations included purulent sputum (84 cases, 74%), chest tightness (49 cases, 43%), and hemoptysis (25 cases, 22%). In addition, a few patients presented symptoms as fever (9 cases, 8%), dyspnea (6 cases, 5%), chest pain (5 cases, 4%), and disturbance of consciousness (1 case, 1%). Physical examinations identified 24 cases (21%) presenting rales and 6 cases (5%) wheezing.

Comparisons in adult patients with radiolucent versus radiopaque FBA were done. A higher proportion of patients without a witnessed choking history was observed in radiolucent FBA cases when compared with those with radiopaque FBA (93% *vs.* 39%; *P* < 0.001). Patients diagnosed with radiolucent FBA also had a longer disease course (median 60 days, IQR 14–150; *P* < 0.001), with 44 cases (51%) having a length of disease course of more than 60 days (*P* = 0.004). 12 (14%) patients diagnosed with radiolucent FBA had been admitted to ICU, while no case was for those with radiopaque FBA (*P* = 0.04). 57 (66%) patients diagnosed with radiolucent FBA had been misdiagnosed as pneumonia compared to 7 (25%) for those with radiopaque FBA (*P* < 0.001). Details are summarized in Table 1.

### Multidetector computed tomography (MDCT) presentations

**Fig. 1:**
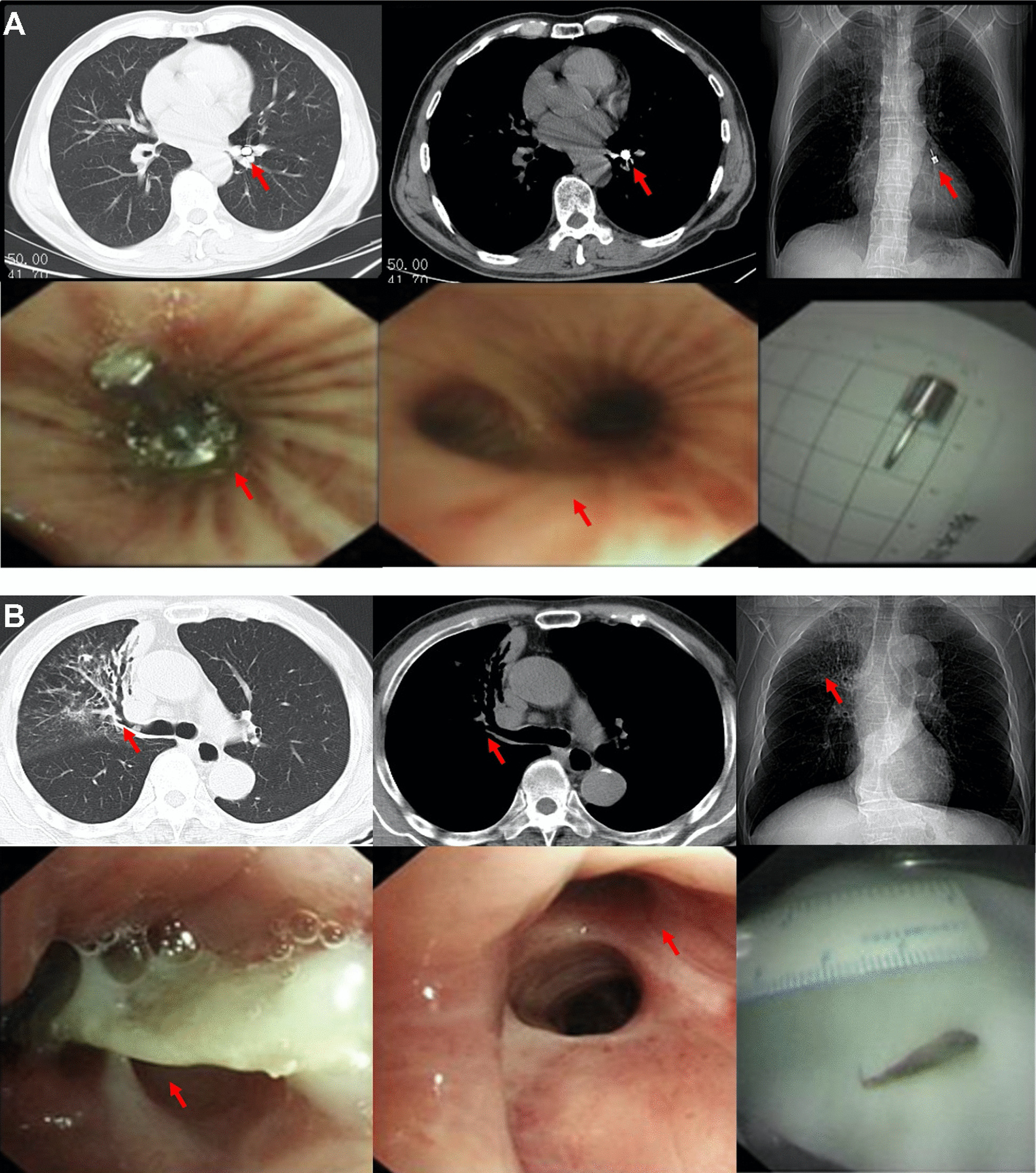
Representative radiographic presentations and bronchoscopic findings from foreign body aspiration cases. **A** A 68-year male patient inhaled a metal nail (red arrows) during dental implantation located at the LB8 in the radiopaque group. **B** A 78-year male patient inhaled a fish bone (red arrows) located at the RB2 in the radiolucent group. In **B**, the position of the fish bone was indicated retrospectively after a flexible bronchoscopy examination

All patients (n = 114) underwent MDCT examinations followed by flexible bronchoscopy, with 28 cases (25%) showing direct visualization of the foreign body (defined as the *radiopaque* group, Fig. 1A) and 86 cases (75%) in the *radiolucent* group (Fig. 1B). In addition to the visualization of the foreign body, MDCT presentations for FBA included non-resolving pneumonia (pneumonic patch) in 80 cases (70%), airway stenosis in 18 cases (16%), atelectasis in 16 cases (14%), bronchiectasis in 15 cases (13%), thickening of the bronchial wall in 14 cases (12%), unilateral pleural effusion in 14 cases (12%), and consolidation in 13 cases (11%) (Table 2).

**Table 2:** Multidetector computed tomography (MDCT) presentations

	All (n = 114)	Radiopaque (n = 28)	Radiolucent (n = 86)	*P-*value
Pneumonic patch	80 (70%)	10 (36%)	70 (81%)	< 0.001*
Airway stenosis	18 (16%)	1 (4%)	17 (20%)	0.07
Atelectasis	16 (14%)	1 (4%)	15 (17%)	0.11
Bronchiectasis	15 (13%)	1 (4%)	14 (16%)	0.11
Thickening of the bronchial wall	14 (12%)	0 (0%)	14 (16%)	0.02*
Unilateral pleural effusion	14 (12%)	1 (4%)	13 (15%)	0.18
Consolidation	13 (11%)	0 (0%)	13 (15%)	0.04*

Data are n (%). *P-value < 0.05 with statistical significance

When comparing adult patients with radiolucent versus radiopaque FBA, we noticed that a higher proportion of patients with radiolucent FBA showed non-resolving pneumonia (pneumonic patch) (81% *vs.* 36%; *P* < 0.001), thickening of the bronchial wall (16% *vs.* 0%; *P* = 0.02) and consolidation (15% *vs.* 0%; *P* = 0.04) (Table 2).

### Flexible bronchoscopy findings

In total, 112 cases (98%) of the foreign bodies were successfully removed by flexible bronchoscopy with 85 cases (75%) achieved by a single operation and 29 cases (25%) needing more than 2 operations. 85 cases (75%) were operated on under local anesthesia and 29 cases (25%) under general anesthesia. Granulomas under bronchoscopy were observed in 50 cases (44%) and ulcers in 9 cases (8%). 85 cases (75%) required a single surgical tool to remove foreign bodies while 29 cases (25%) with more than 2 different types of tools. During operations, 20 cases (18%) presented active bleeding. For operation time, 79 cases (69%) were finished within 30 min and 35 cases (31%) above 30 min. Interestingly, adult patients with radiolucent FBA seemed to require a fewer number of operations (*P* = 0.03) (Table 3).

**Table 3:** Summary of flexible bronchoscopy

	All (n = 114)	Radiopaque (n = 28)	Radiolucent (n = 86)	P-value
Clinical outcome				
Successfully removed	112 (98%)	27 (96%)	85 (99%)	0.43
Unsuccessfully removed	2 (2%)	1 (4%)	1 (1%)	
Number of operations				
< 2	85 (75%)	16 (57%)	69 (80%)	0.03*
≥ 2	29 (25%)	12 (43%)	17 (20%)	
Anesthesia				
Local anesthesia	85 (75%)	25 (89%)	60 (70%)	0.07
General anesthesia	29 (25%)	3 (11%)	26 (30%)	
Manifestations under flexible bronchoscopy				
Granuloma	50 (44%)	8 (29%)	42 (49%)	0.10
Ulcer	9 (8%)	4 (14%)	5 (6%)	0.22
Operation time, minutes				
< 30	79 (69%)	24 (86%)	55 (64%)	0.05
≥ 30	35 (31%)	4 (14%)	31 (36%)	
Number of surgical tools				
≥ 2	29 (25%)	6 (21%)	23 (27%)	0.76
< 2	85 (75%)	22 (79%)	63 (73%)	
Intraoperative complications				
Active bleeding	20 (18%)	5 (18%)	15 (17%)	1

Data are n (%). *P-value < 0.05 with statistical significance

**Fig. 2:**
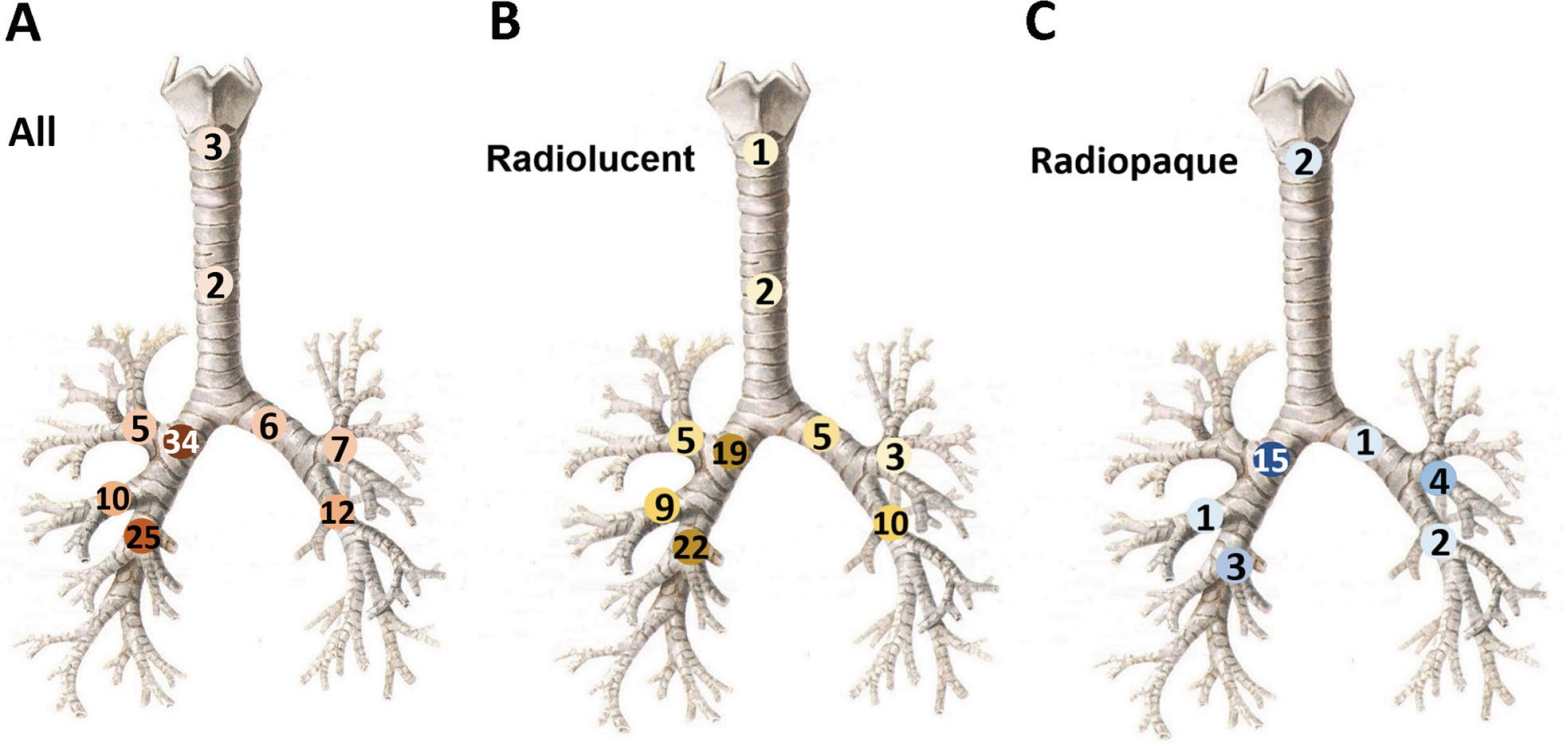
Sites of foreign bodies. Diagrams showing sites of foreign bodies in all cases (**A**, n = 114), the radiolucent group (**B**, n = 86), and the radiopaque group (**C**, n = 28). Numbers in circles are case numbers and the darkness of color represents the frequency

The foreign bodies successfully removed were mostly located in the right main bronchus (34 cases, 30%) and right lower lobe bronchus (25 cases, 22%). Other locations included the left lower lobe bronchus (12 cases, 11%), right middle lobe bronchus (10 cases, 9%), left upper lobe bronchus (7 cases, 6%), left main bronchus (6 cases, 5%), right upper lobe bronchus (5 cases, 4%), glottis (3 cases, 3%), and trachea (windpipe) (2 cases, 2%). Foreign bodies in 10 cases (9%) had multiple locations (Fig. 2A).

A variety of foreign bodies were detected, including 58 cases (51%) of bones (chicken bone, fish bone, crayfish shell), 34 cases (30%) of plants/meat (vegetable, beans, nuts, food scrap mixed with rice, vegetable, and meat), medicine pills (2 cases, 2%), metal (2 cases, 2%), plastic films (2 cases, 2%), and dentures (2 cases, 2%). 11 subjects (10%) were not identifiable (Table 4). We then classified metal, plastic films, and dentures as inorganics, while bones, medicine pills, and plants/meat as organics. In this cohort, 97 cases (85%) were organic foreign bodies (Additional file 1: Table S1).

**Table 4:** Bronchoscopy findings

	All (n = 114)	Radiopaque (n = 28)	Radiolucent (n = 86)	P-value
Site of foreign body				0.005*
Glottis	3 (3%)	2 (7%)	1 (1%)	
Windpipe	2 (2%)	0 (0%)	2 (2%)	
Left main bronchus	6 (5%)	1 (4%)	5 (6%)	
Left upper lobe bronchus	7 (6%)	4 (14%)	3 (3%)	
Left lower lobe bronchus	12 (11%)	2 (7%)	10 (12%)	
Right main bronchus	34 (30%)	15 (53%)	19 (22%)	
Right upper lobe bronchus	5 (4%)	0 (0%)	5 (6%)	
Right middle lobe bronchus	10 (9%)	1 (4%)	9 (10%)	
Right lower lobe bronchus	25 (22%)	3 (11%)	22 (26%)	
Multiple sites	10 (9%)	0 (0%)	10 (12%)	
Type of foreign body				0.03*
Bones	58 (51%)	20 (71%)	38 (44%)	
Medicine pills	2 (2%)	1 (4%)	1 (1%)	
Plants/meat	34 (30%)	3 (11%)	31 (36%)	
Inorganics^#^	6 (5%)	2 (7%)	4 (5%)	
Multiple foreign bodies	3 (3%)	0 (0%)	3 (3%)	
Unknown	11 (10%)	2 (7%)	9 (10%)	

Data are n (%). *P-value < 0.05 with statistical significance. ^#^Metal, plastic films and dentures are classified as inorganics

When comparing adult patients with radiolucent versus radiopaque FBA, we noticed the differences in the site (*P* = 0.005) and type of foreign bodies (*P* = 0.03) (Table 4). The top 2 locations for radiolucent foreign bodies were the right lower lobe bronchus (22 cases, 26%) and right main bronchus (19 cases, 22%) (Fig. 2B), while the majority of radiopaque foreign bodies located at the right main bronchus (15 cases, 53%) (Fig. 2C). Regarding the type of foreign bodies, radiolucent foreign bodies were mainly bones (38 cases, 44%) and plants/meat (31 cases, 36%) (Table 4).

### Risk factors associated with radiolucent foreign body inhalation in adults

To explore the risk factors associated with radiolucent foreign body inhalation in adults, univariate and multivariable logistic regression models were used. In univariate analysis, without a witnessed choking history, a longer disease course, MDCT showing pneumonic patches, located at the right lower lobe bronchus, and plants/meat foreign bodies were associated with radiolucent foreign body inhalation in adults (all *P* < 0.05; Additional file 1: Table S2).

We then identified MDCT showing pneumonic patches (OR 6.99; 95% CI 1.80–27.22; *P* = 0.005) and plants/meat foreign bodies (OR 6.17; 95% CI 1.12–33.96; *P* = 0.04) as independent risk factors of radiolucent foreign body inhalation in adults in the multivariable analysis (Table 5; Additional file 1: Table S3). A witnessed choking history (OR 0.02; 95% CI 0–0.14; *P* < 0.001) was a protective factor of radiolucent foreign body inhalation in adults in this multivariable analysis (Table 5; Additional file 1: Table S3).

**Table 5:** Multivariable stepwise linear regression analysis for risk factors associated with radiolucent FBA in adults

	OR (Odds Ratio)	95% CI (Confidence Interval)	*P-*value
Witnessed choking			
Witnessed choking	0.02	0–0.14	< 0.001*
Multidetector computed tomography (MDCT) presentations			
Pneumonic patch	6.99	1.80–27.22	0.005*
Type of foreign body			
Bones	Ref.	–	–
Medicine pills	2.15	0–1029	0.84
Plants/meat	6.17	1.12–33.96	0.04*
Inorganics^#^	4.81	0.17–133	0.35
Unknown	1.52	0.22–10.21	0.67

^*^P-value < 0.05 with statistical significance. ^#^Metal, plastic films and dentures are classified as inorganics

## Discussion

FBA may present a life-threatening emergency, which requires early diagnosis and urgent removal of foreign bodies by bronchoscopy to avoid complications [9, 12, 25, 26]. However, the presentations and symptoms of FBA in adults are inconsistent and diverse [6, 8–10]. In this retrospective study, with coughing as the most common presentation as expected, other presentations, such as purulent sputum, chest tightness, hemoptysis, and dyspnea, were also recorded. Occasionally, several patients presented symptoms such as fever, chest pain, and disturbance of consciousness.

In addition, foreign body inhalation in adults is often with no direct signs of airway foreign body in chest radiographs [12, 27]. In our cohort, all adult patients underwent MDCT examinations followed by flexible bronchoscopy. There were 75% of patients without direct visualization of the foreign bodies (radiolucent). Within this group, a majority of patients (93%) denied a witnessed choking history. All these factors resulted in a challenging/delayed diagnosis in adults with radiolucent foreign body inhalation. This led to a higher proportion of patients with misdiagnosis, a longer disease course, and being admitted to ICU. These findings were also reflected by the MDCT examinations showing that in the radiolucent group, patients often manifested non-resolving pneumonia (pneumonic patches), thickening of the bronchial wall, and consolidation, indicating recurrent airway inflammation. Taken together, identifying risk factors (predictors) that help in the early diagnosis of radiolucent foreign body inhalation in adults is urgently demanded.

A previous study by Boyd and colleagues suggested that risk factors of FBA in adults include loss of consciousness, age-related slowing in the swallowing mechanism, use of medications (those impair the ability to cough and swallow), post-stroke dysphagia, and numerous neurodegenerative diseases such as Alzheimer's or Parkinson's disease [27]. When comparing adult patients with radiolucent versus radiopaque FBA, no differences were found among the aforementioned risk factors.

To explore the risk factors associated with radiolucent foreign body inhalation in adults, robust and appropriate statistical methods were adopted. We identified MDCT showing pneumonic patches and plants/meat foreign bodies as independent risk factors of radiolucent foreign body inhalation in adults, and a witnessed choking history as a protective factor. This is consistence with earlier discussions indicating that a majority of radiolucent FBA cases in adults show evidence of recurrent airway inflammation as well as lacking a choking event.

Studies showed that the nature of the inhaled foreign bodies is highly variable ranging from organic to inorganic materials in adults [25, 28, 29]. The type of food aspirated varies with local traditions and/or ethnic background [30, 31]. Our study showed that 85% of foreign bodies were organic, including plants/meat and bones from different food. Although chest MDCT is more sensitive to detect radiopaque foreign bodies than X-rays [32], many organic foreign bodies, such as bones and plants/meat are radiolucent under MDCT [33–36]. A study suggested that a plant foreign body is a risk factor for lower respiratory tract infection in children [33]. Some plants, such as nuts, can cause inflammation, granulation tissue formation, and airway stenosis; while beans, seeds, and sweetcorn seeds can absorb water, leading to swelling which causes partial obstruction to a complete obstruction [37]. Multiple medication pills can also cause severe airway inflammation and ulceration [38]. The reason for the false-negative MDCT findings under these conditions could be a consequence of local lung infections resulting in a failed visualization of the foreign body.

The most common site of aspiration in adults is the right bronchus, in particular the mainstem or divisions of the right lower lobe bronchus. This is because compared to the left bronchus, the right one is more vertical and with a slightly larger diameter [39, 40]. This is also true in our cohort, with a majority of foreign bodies located in the right main bronchus and right lower lobe bronchus. Interestingly, the top location for radiolucent foreign bodies is the right lower lobe bronchus compared to the right main bronchus as the top location for radiopaque foreign bodies.

This study has several limitations. As a bronchoscopy and endoscopy center in central China, referral bias can influence the results of this study. Additionally, the details for foreign bodies, such as sharpness, hardness, and stability, were not recorded, which may be associated with different levels of airway inflammation and consequently MDCT presentations. Lastly, interpretation of our findings might be limited by the sample size. Despite these limitations, to our knowledge, this is the largest retrospective study to date on the risk factors associated with radiolucent foreign body inhalation in adults.

## Conclusion

Unlike radiopaque FBA, in those presenting with a suspected radiolucent foreign body aspiration, the diagnosis is far more challenging. This study identifies a few risk factors that have the potential to facilitate an early diagnosis of radiolucent foreign body inhalation in adults. Further prospective multicenter studies should be conducted to validate the findings.

## Supplementary Information

**Additional file 1: Methods.** Flexible bronchoscopy. **Table S1.** Bronchoscopy findings. **Table S2.** Univariate logistic regression analysis for risk factors associated with radiolucent FBA in adults. **Table S3.** Multivariable logistic regression analysis for risk factors associated with radiolucent FBA in adults.

## Data Availability

The data that support the findings of this study are available from Xioafan Liu upon reasonable request and with permission of The Central Hospital of Wuhan, Hubei, China.

## Abbreviations

FBA: Foreign body aspiration
MDCT: Multi-detector Computed Tomography
