# Supplementary material for: Risk factors associated with radiolucent foreign body inhalation in adults: a 10-year retrospective cohort study
Source: Respir Res. Author manuscript; Available in PMC 2023 Oct 12. (PMC9463778; doi:10.1186/s12931-022-02165-9)
Supplement: Supplementary Methods [file EMS154139-supplement-Supplementary_Methods.pdf]

Supplementary Methods

Flexible bronchoscopy

Flexible bronchoscopy was undertaken following "guideline for diagnostic flexible bronchoscopy (2008)"[1] and "guideline for diagnostic flexible bronchoscopy in adults (2019)"[2]. In brief, flexible bronchoscopy was offered under the following conditions, *e.g.* a witnessed choking history, refractory or non-resolving pneumonia, suspected lung cancer, CT imaging presentations showing airway stenosis, obstructive pneumonia, and atelectasis.

Local or general anesthesia was applied before undergoing flexible bronchoscopy. All adults were operated on via flexible bronchoscopy (Olympus / Pentax 6.0 mm with working channel 2.8 mm, and 6.1 mm with working channel 3.2 mm) after 6 h of fasting. Written informed consent for performing bronchoscopy was provided before the procedure. The surgical tools to remove foreign bodies included foreign-body forceps, biopsy forceps, snares, foreign-body baskets, balloon catheters, cryoprobes, and LASER. Forceps are used to hold nonfriable objects like coins, bones, and plastics, and are usually not used to retrieve friable objects like food and nuts as grasping them with forceps leads to their breakdown. Snares and baskets are useful for the removal of flat or elongated objects or large and friable objects. A balloon catheter can be used for FBs impacted in the distal airways, compression bleeding airway mucosa after retrieval of foreign body, or restrict the blood in operation entering another airway with presetting. Foreign bodies that contain high water content can be handled with cryoprobe for freezing and adhering. When the FB is embedded in the airway wall may need to be broken down into smaller parts by using LASER techniques before being extracted from the airway. Further, LASER is used for electrocoagulation hemostasis on blood small vessel.

After the removal of foreign bodies, another bronchoscopy was performed to check for any remains, assess any injuries to the airway, remove post-obstructive secretions or pus, control any active bleeding and remove granulation tissue that may obstruct the airway.

References:

1. Interventional pulmonology group of the Chinese Thoracic Society, Chinese Medical Association. (2008). [Guideline for diagnostic flexible bronchoscopy (2008)]. *Zhonghua Jie He He Hu Xi Za Zhi (Chinese Journal of Tuberculosis and Respiratory Diseases)*, 31(1), 14-17.

2. Interventional pulmonology group of the Chinese Thoracic Society, Chinese Medical Association. (2019). [Guideline for diagnostic flexible bronchoscopy in adults (2019)]. *Zhonghua Jie He He Hu Xi Za Zhi (Chinese Journal of Tuberculosis and Respiratory Diseases)*, 42(8), 573-590. doi:10.3760/cma.j.issn.1001-0939.2019.08.005

Supplementary Tables

Supplementary Table 1: Bronchoscopy findings.

Supplementary Table 2: Univariate logistic regression analysis for risk factors associated with radiolucent FBA in adults.

Supplementary Table 3: Multivariable logistic regression analysis for risk factors associated with radiolucent FBA in adults.

Supplementary Table 1: Bronchoscopy findings.

	**All (n=114)**	**Radiopaque (n=28)**	**Radiolucent (n=86)**	***P*-value**
**Type of foreign body**				0.80
Unknown	11 (10%)	2 (7%)	9 (10%)	
Inorganics	6 (5%)	2 (7%)	4 (5%)	
Organics	97 (85%)	24 (86%)	73 (85%)	

Data are n (%). Metal, plastic films, and dentures are classified as **inorganics**; while bones, medicine pills, and plants/meat as **organics**.

Supplementary Table 2: Univariate logistic regression analysis for risk factors associated with radiolucent FBA in adults.

	**OR (Odds Ratio)**	**95% CI (Confidence Interval)**	***P*-value**
**Length of disease course, days**			
≥ 60	4.82	1.68 ~ 13.85	0.003*
**Witnessed choking**			
Witnessed choking	0.02	0 ~ 0.08	< 0.001*
**Multidetector computed tomography (MDCT) presentations**			
Pneumonic patch	7.87	3.06 ~ 20.26	< 0.001*
Thickening of the bronchial wall	16545204	0 ~ ∞	0.99
Consolidation	16318558	0 ~ ∞	0.99
**Site of foreign body**			
Right main bronchus	Ref	-	-
Glottis	0.39	0.03 ~ 4.78	0.47
windpipe	91301679	0 ~ ∞	1.00
Right upper lobe bronchus	91301678	0 ~ ∞	0.99
Right middle lobe bronchus	7.11	0.81 ~ 62.49	0.08
Right lower lobe bronchus	5.79	1.45 ~ 23.09	0.01*
Left upper lobe bronchus	0.59	0.11 ~ 3.06	0.53
Left lower lobe bronchus	3.95	0.75 ~ 20.81	0.11
Left main bronchus	3.95	0.42 ~ 37.5	0.23
Multiple sites	91301678	0 ~ ∞	0.99
**Type of foreign body**			
Bones	Ref	-	-
Medicine pills	0.53	0.03 ~ 8.87	0.66
Plants/meat	5.44	1.48 ~ 20.01	0.01*
Inorganics	1.05	0.18 ~ 6.25	0.96
Unknown	2.37	0.47 ~ 12.03	0.30
**Type of foreign body**			
Unknown	Ref	-	-
Inorganics	0.44	0.05 ~ 4.37	0.49
Organics	0.68	0.14 ~ 3.35	0.63

P*-value < 0.05 with statistical significance. Metal, plastic films, and dentures are classified as **inorganics; while bones, medicine pills, and plants/meat as **organics**.

Supplementary Table 3: Multivariable logistic regression analysis for risk factors associated with radiolucent FBA in adults.

	OR (Odds Ratio)	95% CI (Confidence Interval)	*P*-value
**Multivariable**			
**Length of disease course, days**			
≥ 60	1.45	0.29 ~ 7.35	0.65
**Witnessed choking**			
Witnessed choking	0.004	0 ~ 0.09	< 0.001*
**Multidetector computed tomography (MDCT) presentations**			
Pneumonic patch	5.57	0.80 ~ 38.85	0.08
**Site of foreign body**			
Right main bronchus	Ref	-	-
Glottis	0.02	0 ~ 1.59	0.08
Windpipe	1697224518	0 ~ ∞	1
Right upper lobe bronchus	8128098	0 ~ ∞	1
Right middle lobe bronchus	12.07	0.27 ~ 539.14	0.20
Right lower lobe bronchus	4.46	0.38 ~ 52.98	0.24
Left upper lobe bronchus	0.62	0.06 ~ 5.95	0.68
Left lower lobe bronchus	0.53	0.07 ~ 4.18	0.54
Left main bronchus	22.20	0.07 ~ 6875.41	0.29
Multiple sites	1419094	0 ~ ∞	1
**Type of foreign body**			
Bone	Ref	-	-
Medicine pill	3.28	0 ~ 1885227	0.86
Plants/meat	17.89	1.09 ~ 292.83	0.04*
Inorganics#	38.42	0.43 ~ 3399	0.11
Unknown	1.17	0.13 ~ 10.97	0.89
**Multivariable (after stepwise regression)**			
**Witnessed choking**			
Witnessed choking	0.004	0 ~ 0.09	< 0.001*
**Multidetector computed tomography (MDCT) presentations**			
Pneumonic patch	6.52	1.03 ~ 41.13	0.046*
**Site of foreign body**			
Right main bronchus	Ref	-	-
Glottis	0.02	0 ~ 1.42	0.07
Windpipe	1457062079	0 ~ ∞	1.00
Right upper lobe bronchus	9729174	0 ~ ∞	1.00
Right middle lobe bronchus	11.5	0.29 ~ 458	0.19
Right lower lobe bronchus	4.35	0.37 ~ 50.37	0.24
Left upper lobe bronchus	0.58	0.06 ~ 5.57	0.64
Left lower lobe bronchus	0.54	0.07 ~ 4.30	0.56
Left main bronchus	25.14	0.10 ~ 6295	0.25
Multiple sites	1206074	0 ~ ∞	1.00

**Type of foreign body**			
Bone	Ref	-	-
Medicine pill	3.67	0 ~ 1191402	0.84
Plants/meat	19.15	1.13 ~ 326	0.04*
Inorganics	48.72	0.65 ~ 3678	0.08
Unknown	1.27	0.14 ~ 11.76	0.83

P*-value < 0.05 with statistical significance. Metal, plastic films, and dentures are classified as **inorganics.
